# piRNAs may regulate expression of candidate genes of esophageal adenocarcinoma

**DOI:** 10.3389/fgene.2022.1069637

**Published:** 2022-11-30

**Authors:** A. N. Akimniyazova, T. K. Niyazova, O. Yu. Yurikova, A. Yu. Pyrkova, M. A. Zhanuzakov, A. T. Ivashchenko

**Affiliations:** ^1^ Higher School of Medicine, Faculty of Medicine and Healthcare, Al-Farabi Kazakh National University, Almaty, Kazakhstan; ^2^ Department of Biotechnology, Faculty of Biology and Biotechnology, Al-Farabi Kazakh National University, Almaty, Kazakhstan; ^3^ Center for Bioinformatics and Nanomedicine, Almaty, Kazakhstan

**Keywords:** esophageal adenocarcinoma, piRNA, mRNA, candidate genes, diagnostic

## Abstract

Elucidation of ways to regulate the expression of candidate cancer genes will contribute to the development of methods for cancer diagnosis and therapy. The aim of the present study was to show the role of piRNAs as efficient regulators of mRNA translation of esophageal adenocarcinoma (EAC) candidate genes. We used bioinformatic methods to study the interaction characteristics of up to 200 thousand piRNAs with mRNAs of 38 candidate EAC genes. The piRNAs capable of binding to mRNA of *AR, BTG3, CD55, ERBB3, FKBP5, FOXP1, LEP, SEPP1, SMAD4,* and *TP53* genes with high free energy by the formation of hydrogen bonds between canonical (G-C, A-U) and noncanonical (G-U, A-C) piRNA and mRNA nucleotide pairs were revealed. The organization of piRNA binding sites (BSs) in the mRNA of candidate genes was found to overlap nucleotide sequences to form clusters. Clusters of piRNA BSs were detected in the 5′-untranslated region, coding domain sequence, and 3′-untranslated region of mRNA. Due to the formation of piRNA binding site clusters, compaction of BSs occurs and competition between piRNAs for binding to mRNA of candidate EAC genes occurs. Associations of piRNA and candidate genes were selected for use as markers for the diagnosis of EAC.

## Introduction

The biological role of piRNA (PIWI-interacting RNA), despite the long-standing discovery of piRNA (Kim, 2006; Aravin et al., 2007), remains at the stage of speculation. There are no works on the direct interaction of piRNAs with mRNAs of genes. In recent years, there have been reports of the involvement of piRNA in the regulation of many biological processes (Czech et al., 2018; Chen et al., 2021; Zhang et al., 2022a). Since the present work is devoted to the involvement of piRNA in carcinogenesis, here we will review the existing ideas about the role of piRNA in the development of various forms of cancer. It has been shown that piRNAs are abnormally expressed in various forms of cancer and the authors suggest using piRNA in diagnosis and therapy (Liu et al., 2019). In recent years, the role of piRNA in the development of breast cancer has been actively studied. The molecular mechanisms and possible clinical implications of PIWI-interacting RNA have been investigated in this disease (Tan et al., 2019; Qian et al., 2021; Yuan et al., 2021). PIWI-interacting RNAs have been shown to be involved in proliferation, migration, and apoptosis in breast cancer (Kärkkäinen et al., 2021; Liu et al., 2021). The role of piRNA in the pathogenesis and diagnosis of esophageal cancer, colon and stomach cancer, colorectal cancer has been considered (Lin et al., 2019; Halajzadeh et al., 2020; Ameli Mojarad et al., 2022; Jia et al., 2022). The biogenesis and role of piRNA in several cancer localizations at once have been studied (Guo et al., 2020). A number of studies suggest that piRNAs are involved in oncogenesis through their influence on genes, but how this occurs remains unknown (Fonseca et al., 2020; Wang et al., 2022a; Zhang et al., 2022a; Zhang et al., 2022b; Cai et al., 2022; Zhang et al., 2022c; Hanusek et al., 2022). In recent years, the study of piRNAs has continued to receive a great deal of attention. For example, it has been shown that out of 902 piRNAs expressed in somatic cells 527 are synthesized in the brain, of which piR-hsa-92056, piR-hsa-150797, piR-hsa-347751, piR-hsa-1909905, piR-hsa-2476,630, and piR-hsa-2834636 are recommended as biomarkers for Parkinson’s disease (Zhang and Wong, 2022).

Published articles showing the important role of piRNA in gastrointestinal cancer (Lin et al., 2019; Mojarad et al., 2022). The piRNAs dysregulations were reported to promote or suppress the initiation and development of different malignancies, especially gastrointestinal cancers. Recently, suggested the use of piRNAs as potential cancer biomarkers associated with the progression and chemoresistance of gastrointestinal cancer. Hence, this review article aims to focus on the role of piRNAs in gastrointestinal cancer progression, metastasis, and their molecular mechanisms as therapeutic markers for gastrointestinal cancer patients. X. Lin et al., 2022 identified 8,759 piRNAs in the human stomach. Of these, about 50 piRNAs could be differentially expressed compared to controls. It has been shown that piRNA/PIWI protein-mediated DNA methylation regulation mechanisms and methylation changes caused by piRNA/PIWI proteins in different diseases, especially cancers (Jia et al., 2022). Several studies suggest piRNA**s** as potential cancer biomarkers. Translational studies suggest that piRNA**s** may regulate key genes and pathways associated with gastric cancer progression, though there is no functional annotation in piRNA (Fonseca et al., 2020). Previously, 6,260 human piRNAs transcriptomes derived from benign and tumor tissues were analyzed, of which 522 piRNAs are expressed in the corresponding tumor tissues (Martinez et al., 2015). The authors suggest that piRNA expression may determine clinical features such as histological subgroups, disease stages, and survival. The piRNAs common to many cancer types may represent a core set of genes that contribute to cancer growth, while piRNAs unique to individual cancer types are likely specific.

The strongest stimulus for the study of the biological role of piRNA was the creation of an extensive database of piRNA properties (Wang et al., 2019; Wang et al., 2021). The publication of changes in piRNA synthesis during ontogenesis has made a significant contribution to the study of piRNA (Zhou et al., 2018). Recently, it was shown that piRNAs can bind to mRNAs of protein-coding genes involved in the development of various diseases (Belkozhayev et al., 2022). This information prompts us to investigate how and which piRNAs can regulate the expression of protein-coding genes. This work aims to show the possibility of the significant influence of piRNAs on the translation process through the interaction of these molecules with mRNAs of candidate genes involved in the development of esophageal adenocarcinoma (EAC).

## Materials and methods

The nucleotide sequences of candidate genes of EAC were downloaded from NCBI. The nucleotide sequences of the first 200 thousand piRNAs of the 8,400,000 piRNAs database were taken from Wang et al. (Wang et al., 2021). The piRNA binding sites (BSs) in the mRNAs of genes were predicted using the MirTarget program (Ivashchenko et al., 2014). This program defines the following features of piRNA binding to mRNA: a) the initiation of piRNA binding from the first nucleotide of the mRNAs; c) the schemes of nucleotide interactions between piRNAs and mRNA; d) the free energy ΔG (kJ/mol) of the interaction between piRNA and the mRNA; e) the ratio ΔG/ΔGm (%) is determined for each site (ΔGm equals the free energy of piRNA binding with its fully complementary canonical nucleotide sequence). Only piRNAs whose nucleotides interacted with mRNA through canonical (G-C and A-U) and noncanonical (G-U and A-C) nucleotides with a ΔG/ΔGm value of 90% or more were selected from the calculated data. The 5′-untranslated region (5′UTR), coding domain sequence (CDS), and the 3′-untranslated region (3′UTR) of the mRNAs; c) the schemes of nucleotide interactions between miRNAs, piRNAs and mRNAs d) the free energy of the interaction between miRNAs, piRNAs and the mRNA (ΔG/ΔGm, %); and the ratio ΔG/ΔGm is determined for each site. The MirTarget program finds hydrogen bonds between adenine (A) and uracil (U), guanine (G) and cytosine (C), G and U, and A and C. Regarding the free energy of interactions (∆G), a pair of G and C is equal to 6.37 kJ/mol, a pair of A and U is equal to 4.25 kJ/mol, and a pair of G and U or A and C is equal to 2.12 kJ/mol. The distances between bound A and C (1.04 nm) and G and U (1.02 nm) are similar to those between bound G and C and between bound A and U and are equal to 1.03 nm (Friedman and Honig, 1995; Garg and Heinemann, 2018). The numbers of hydrogen bonds in the G–C, A–U, G–U, and A–C interactions were 3, 2, 1, and 1, respectively. By comparison, MirTarget differs from STarMir Tools, miRWalk programs (Kanoria et al., 2016; Sticht et al., 2018) in terms of finding the BSs of piRNA on the mRNAs in the following: (Kim, 2006): it accounts for the interaction of the piRNAs with mRNA over the entire piRNAs sequence; (Aravin et al., 2007); it considers noncanonical nucleotide pairs; and (Czech et al., 2018) it calculates the free energy of the interaction of the piRNAs with mRNA. When two or more piRNAs are bound with one mRNA, or if the BSs of two different piRNAs coincide in part, the preferred piRNA BS is considered to be the one for which the free energy binding is greater (Leontis et al., 2002; Davis et al., 2005).

## Results

Androgen receptor (*AR*) is one of 38 candidate EAC genes (Bahramian et al., 2022) (Supplementary Table S1). Of the 200 thousand piRNAs we studied, only piR-32860 could bind to the mRNA of the *AR* gene. A feature of piR-32860 is the presence of two regions of BSs in the CDS mRNA located with overlapping nucleotide sequences, which we called clusters (Supplementary Table S2). The 15 piR-32860 BS starts at 1,287 nt and arranged sequentially through three nucleotides. They form a cluster whose nucleotides (CAG_22_) encode a polyglutamine of 23 amino acid residues. This triplet arrangement allows three piR-32860 molecules to bind to the mRNA simultaneously. The second cluster of BSs for piR-32860 is located in the CDS mRNA at 2,486 nt (Supplementary Table S2) and encodes for polyglycine. Consequently, several piR-32860 can simultaneously bind to the mRNA of the gene and significantly inhibit AR protein synthesis. The first cluster of BSs with a length of 67 nt is 5.4 times shorter than the total length of 15 piRNAs. The second cluster with a length of 49 nt is 4.4 times shorter than the total length of nine piRNAs. Such compaction of the BSs allows us to reduce significantly the length of the BSs in the coding region so that the rest of the CDS can vary and modify the functional activity of the *AR* gene.

The EAC suppressor protein BTG3 (Du et al., 2015) is encoded by an mRNA region located from 261 nt to 1,151 nt. The starting BSs of 27 piRNAs are located in the CDS mRNA from 573 nt to 665 nt with overlapping nucleotide sequences of piRNAs (Supplementary Table S3). The BSs of piR-12399, piR-37172, piR-126527, and piR-129369 start at 626 nt, which is approximately in the middle of the first BSs. The BSs cluster length of 27 piRNAs is 118 nt and is 7.6 times shorter than the CDS length and 135 times shorter than the entire mRNA length. Consequently, maintaining the nucleotide conserved nature of the cluster of piRNAs BSs requires a relatively small fraction of the mRNA nucleotide sequence to maintain the dependence of gene expression on 27 piRNAs. The second cluster of BSs of 11 piRNAs is located in the 3′UTR from 1,420 nt to 1,485 nt long by 66 nt and is 72.7 times shorter than the 3′UTR and 236 times shorter than the entire mRNA. This distribution of piRNAs BSs in the mRNA is not random, as it is organized into clusters. The piR-89068 and piR-83600, with a length of 34 nt, bind to mRNA with a ΔG value of −178 kJ/mol and −172 kJ/mol, respectively, indicating a high dependence of gene expression on these piRNAs. The binding of piR-75778 to mRNA occurs by forming almost only canonical nucleotide pairs, as the ΔG/ΔGm value is 99% (Supplementary Table S3). The piRNAs from piR-12399 to piR-129369, i.e., almost the entire interval of 200 thousand piRNAs, bind to the mRNA of the *BTG3* gene. The distribution of piRNAs BSs in the form of clusters allows, firstly, compactification of BSs and, secondly, competition for binding to mRNA arises between piRNAs. The piRNA that interacts more strongly with the mRNA or the piRNA that is present in a much higher concentration compared to other piRNAs will predominantly bind to the mRNA.

Eleven piRNA BSs were identified in the mRNA CDS of the CD55 gene, located from 295 nt to 1,617 nt, with an origin from 1,420 nt to 1,458 nt (Supplementary Table S4). That is, the length of the cluster of piRNA BSs is 63 nt, which is 21 times shorter than the length of CDS. Among all the piRNAs, piR-83600 stands out, which binds to the mRNA of the *CD55* gene with a free energy of −172 kJ/mol and a ΔG/ΔGm value of 92%. It is very likely that this piRNA can suppress the expression of the candidate *CD55* gene associated with the development of EAC (Murao et al., 2016).

Expression of the *ERBB3* gene (Yoon et al., 2014) can depend on 26 piRNAs, the BSs of which are located in the 3′UTR from 4,940 nt to 5,097 nt (Supplementary Table S5). These piRNA BSs formed a cluster from 4,940 nt to 5,125 nt, 186 nt long. The 3′UTR was 7.8 times as long as the BSs cluster. A feature of this cluster is the presence of an identical BS for seven piRNAs: piR-5300, piR-5303, piR-5358, piR-6236, piR-7637, piR-65119, piR-96686, each of which interacted with mRNA to approximately the same extent (Supplementary Table S5). The second cluster consisted of the BSs of nine piRNAs (Supplementary Table S5). The cluster length of 35 nt was 41.5 times shorter than the length of the 3′UTR.

The mRNA of the *FKBP5* gene (Smith et al., 2016) contained five clusters of piRNA BSs located in 3′UTR the mRNA (Supplementary Table S6). In the first cluster, 20 piRNAs bound from 1,382 nt to 1,422 nt in the length of 72 nt. The second cluster starts from 4,854 nt (piR-123782) to 4,875 nt (piR-136223) and binds 20 piRNAs. This cluster contains five BSs with the same start for piRNA at position 4,862 nt (piR-56610, piR-62204, piR-75328, piR-92776, piR-102326). The ten piRNAs have BSs in the third BSs cluster at 4,940 nt to 4,965 nt. The fourth BSs cluster starts from 6,356 nt to 6,578 nt and contains 88 piRNA BSs. The BSs for 116 piRNAs start at position 7,100 nt to 7,201 nt. The piR-74093, piR-75328, piR-91782, piR-36610, piR-115392, piR-110734, piR-35260, piR-94289, piR-123273, piR-95238. piR-37678, piR-123924, piR-65906, piR-120553, piR-79631, piR-98746, piR-83452, piR-89965 binds with mRNA of *FKBP5* gene with ΔG value from −170 kJ/mol to −183 kJ/mol. Of these, piR-65906 binds with a ΔG/ΔGm value of 98%, which means that almost all of it interacts through canonical nucleotide pairs. The piR-45567, piR-59915, piR-56610, piR-91782 bind with a value of ΔG/ΔGm equal to 99%, which also indicates their effectiveness on protein synthesis. Consequently, these piRNAs have the greatest ability to regulate *FKBP5* gene expression. The piRNAs from piR-1248 to piR-198479 bind to the mRNA of *FKBP5* gene, i.e., practically from the whole range of 200 thousand piRNAs studied. The total length of all clusters is 684 nt and is 9.4 times shorter than the entire 3′UTR region and 10.8 times shorter than the entire mRNA. Nine piRNAs (piR-5300, piR-5303, piR-5358, piR-6236, piR-7637, piR-35905, piR-65119, piR-96686, and piR-101606) from the third cluster bound at one position 7,115 nt (Supplementary Table S6). A group of five piRNAs (piR-45567, piR-93145, piR-96134, piR-123755, and piR-169382) bound at position 7,167 nt.

Expression of the *LEP* gene (Bain et al., 2014) may also depend on piRNAs, because 162 piRNAs can bind to only 3′UTR mRNA, including piR-23387, which is completely complementary due to canonical nucleotides (Supplementary Table S7). The BSs of 51 piNAs formed a cluster from 3,084 nt to 3,127 nt located in the 3′UTR. The length of the BSs cluster was 41 times less than the length of the 3′UTR. A group of nine piRNAs (piR-5300, piR-5303, piR-5358, piR-6236, piR-7637, piR-35905, piR-65119, piR-96686, and piR-101606) with BSs with 3,101 nt coincides with a group of piRNAs that bind to the mRNA of the *FKBP5* gene and includes a group of piRNAs that interact with the mRNA of the *ERBB3* gene. The mRNA of the gene contains BSs for three groups each of five piRNAs at positions 3,087 nt, 3,088 nt, and 3,246 nt piR-23387, piR-5299 and piR-1748 interacted with mRNA with ΔG/ΔGm equal to 99% and 100%.

The 46 piRNAs bind to the mRNA of the *SEPP1* gene (Takata et al., 2012) from 108 nt to 172 nt (65 nt) at a 5′UTR length of 188 nt, i.e., 2.9 times shorter (Supplementary Table S8). The length of all 46 binding piRNAs is 1,367 nt, and due to the compaction of piRNAs BSs into clusters, competition between piRNAs for binding in the cluster simultaneously arises. At position 123 nt of the 5′UTR of the mRNA of the *SEPP1* gene, a group of nine piRNAs was identified, identical to the groups of piRNAs that bind to the mRNA of the *ERBB3, FKBP5* and *LEP* genes.

The 74 piRNAs bound to the 3′UTR of the *SMAD4* gene (Weaver et al., 2014). The mRNA and their BSs formed clusters from 4,316 nt to 4,563 nt (Supplementary Table S9). In the cluster, three groups of piRNAs were identified. The first group from 4,335 nt included piR-36976, piR-50827, piR-84920, piR-116971, piR-125671. The second group from 4,337 nt consisted of piR-19014, piR-19076, piR-82088, piR-125598. The third group from 4,401 nt was formed by piR-56309, piR-81517, piR-98951. The cluster was only 247 nt from the 3′UTR length of 6,576 nt, which is 26.6 times the length of the cluster. The piR-82439, piR-84833, piR-100395 interacted with mRNA with a ΔG/ΔGm value of 99%.

There were no clusters of BSs in the mRNA of the *TP53* gene (Fisher et al., 2017) for groups with 5 piRNAs or more (Supplementary Table S10). The cluster of 27 piRNAs BSs with a length of 224 nt was 6.3 times less than the 3′UTR length of 1,408 nt. The piR-44059 and piR-83728 bound to the mRNA of the *TP53* gene with ΔG kJ/mol equal to -170 kJ/mol and 172 kJ/mol, respectively, i.e., they could strongly inhibit the translation process.

**FIGURE 1:**
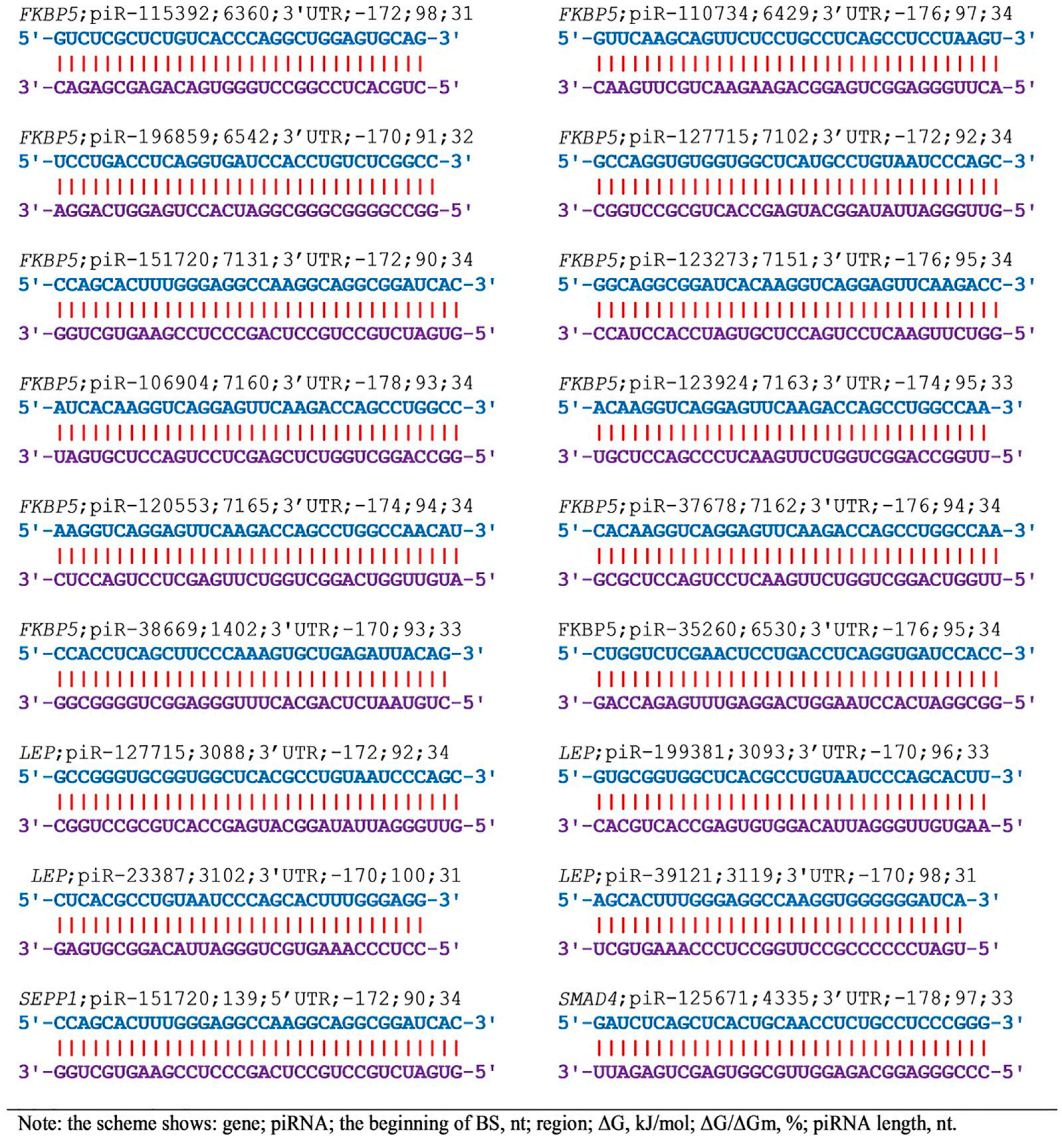
The schemes of interaction between piRNAs and mRNAs of EAC candidate genes with ΔG values of −170 kJ/mol and more.

Based on these studies, the associations of piRNA and candidate genes effectively interacting with a ΔG value of -170 kJ/mol or more are proposed as disease markers. Figure 1 shows the interaction patterns of piRNA with mRNA of candidate EAC genes with a ΔG value of -170 kJ/mol or more. No piRNAs with this interaction energy have been identified for the *AR* gene (Supplementary Table S2). The piR-89068, piR-83600 bind to the CDS mRNA of the *BTG3* gene. Only piR-83600 interacts with the CDS mRNA of the *CD55* gene. Consequently, piR-83600 can regulate the expression of *BTG3* and *CD55* genes. These findings show the need to study piRNA and gene associations in diseases specifically, and not just piRNA or gene associations due to the ambiguity of interpreting results separately for piRNA or genes.

**FIGURE 2:**
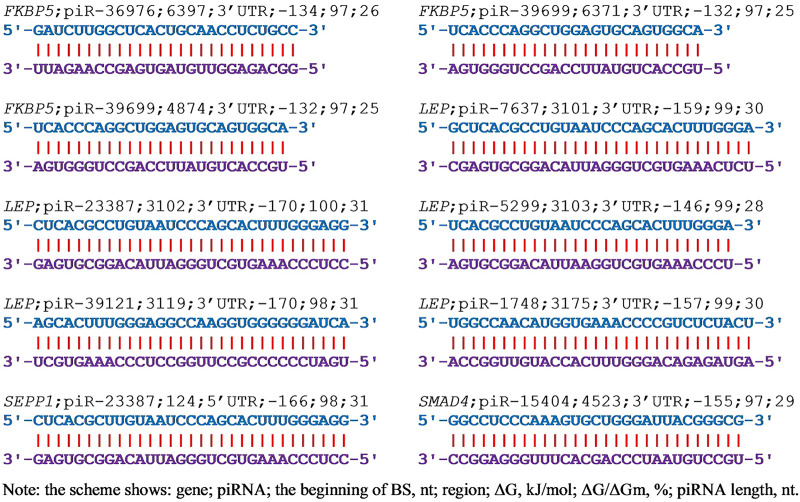
The schemes of interaction between piRNAs and mRNAs of EAC candidate genes with ΔG/ΔGm) values greater than 97% or more.

In selecting piRNAs as markers of a disease caused by a candidate gene, it is necessary to know how selectively the piRNA alters the expression of that gene. Therefore, it is necessary to establish how this (these) piRNAs can change the expression of other genes. We used 200 thousand piRNAs to identify their interactions with the mRNA of 38 candidate EAC genes (Supplementary Table S1) and showed that only nine genes were targeted by the studied piRNAs. From two piRNAs (*AR* gene) to 254 piRNAs (*FKBP5* gene) that bind to mRNA with a ΔG/ΔGm value of 90% or more could interact with these genes. Increasing the selection criterion for piRNAs (ΔG/ΔGm ratio) from 90% to 95% reduces the number of interacting piRNAs by about three times. In cells, the concentration of piRNAs can vary by several orders of magnitude, then a piRNA interacting is weaker than another piRNA but present in a higher concentration will more effectively suppress the expression of the candidate gene. Therefore, we selected piRNAs with a ΔG/ΔGm value of 90% or higher to account for piRNAs potentially being able to bind to the mRNA of the candidate gene. Figure 2 shows as an example the interaction schemes of piRNAs with the mRNA of candidate EAC genes with ΔG/ΔGm values of more than 97% or higher.

This raises the question of whether the free energy of the interaction of piRNA with mRNA (ΔG) or the degree of complementarity of the binding of piRNA to mRNA (ΔG/ΔGm) is a more important factor in assessing the interaction of piRNA with mRNA. Interestingly, the group of piRNA selected on the principle of free energy greater than -170 kJ/mol and the group of piRNA associating with mRNA with ΔG/ΔGm greater than 90% tends to have different clusters of piRNA BSs, which demonstrates the importance of both criteria for the interaction of piRNA with mRNA.

An important aspect of the choice of associations of piRNAs and candidate genes is that one gene may be involved in the development of several diseases and then that gene, regulated even by one piRNA, may be associated by side effects with another disease. Modern drugs come with annotations about the limitations and side effects of their use. Similarly, piRNAs chosen for diagnosis and therapy should be accompanied by a similar annotation. Therefore, laboratory studies of the proposed piRNAs in combination with candidate genes for these diseases are necessary to the evaluative bioinformatics studies of the involvement of piRNAs in various diseases.

The highest number of piRNAs were associated with a ΔG value of −170 kJ/mol or more with the 3′UTR mRNA of the *FKBP5* gene: piR-74093, piR-38669, piR-75328, piR-56610, piR-115392, piR-110734, piR-35260, piR-55529, piR-94289, piR-196859, piR-127715, piR-89432, piR-151720, piR-123273, piR-106904, piR-95238, piR-37678, piR-123924, piR-65906, piR-120553, piR-79631, piR-98746, piR-83452, and piR-89965. Six piRNAs with ΔG values ranging from -170 kJ/mol to -183 kJ/mol bind from 7,160 nt to 7,165 nt in the 3′UTR mRNA of the *FKBP5* gene. This indicates that these piRNAs compete with each other and their suppressive effect on FKBP5 protein synthesis is high.

The piR-127715, piR-89432, piR-199381, piR-23387, piR-39121, piR-97487, piR-73272, piR-77917, piR-78890, piR-87066 can bind to 3′UTR mRNA of *LEP* gene with ΔG value of -170 kJ/mol or more. Note that piR-73272, piR-77917, and piR-78890 bound to the mRNA of the *LEP* gene with a ΔG value of −183 kJ/mol, −183 kJ/mol, and −189 kJ/mol, respectively, with overlapping nucleotide sequences of BSs. Consequently, these piRNAs cannot bind simultaneously, but their overall effect is high, even if they are present in the cell at different concentrations.

The piR-89432 and piR-151720 binded to a ΔG value of -170 kJ/mol or more in the 5′UTR mRNA of the *SEPP1* gene. The piR-125671, piR-82439, piR-125598, piR-98281, piR-94289, piR-83728, and piR-58129 interacted with the 3′UTR mRNA of the *SMAD4* gene with a ΔG value of −170 kJ/mol or more. Only piR-83728 and piR-44059 bind to the 3′UTR mRNA of the *TP53* gene with a free energy of -172 kJ/mol and −170 kJ/mol, respectively. These results demonstrate the involvement of piR-83728 in the regulation of *SMAD4* and *TP53* gene expression. This is another example of the need to study piRNA and gene associations rather than individual piRNAs or genes.

We identified piR-12340, piR-48382, piR-48873, piR-55529, piR-59915, piR-96587, piR-99501, and piR-110973 having BSs in mRNA clusters of *FKBP5* and *SMAD4* genes. Consequently, these piRNAs can simultaneously regulate the expression of *FKBP5* and *SMAD4* genes.

**FIGURE 3:**
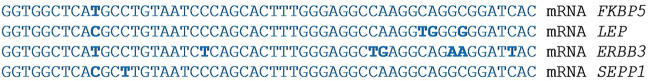
Nucleotide sequences of 27 piRNAs clusters of BSs in the 3′UTR mRNA of the *ERBB3, FKBP5,* and *LEP* genes and in the 5′UTR mRNA of the *SEPP1* gene.

An even more surprising result was obtained with the *ERBB3*, *FKBP5*, *LEP*, and *SEPP1* genes. The mRNAs of these genes contain highly homologous BSs region of 27 piRNAs 54 nt long (Figure 3, (Supplementary Table S11). These data show that the expression of the *ERBB3, FKBP5, LEP,* and *SEPP1* genes can be maintained in a balanced manner by this number of piRNAs. We obtained a similar result when we studied the action of several miRNAs on a number of candidate atherosclerosis genes (Mukushkina et al., 2020).

## Discussion

Many human genes are functionally related, so a single gene can exert its effect in several diseases. For example, the *FKBP5* gene encodes a Prolyl isomerase that binds to immunosuppressants and interacts functionally with a number of proteins. Changes in *FKBP5* gene expression were found in gastric cancer (Kang et al., 2012; Wang et al., 2022b), pancreatic cancer (Hou and Wang, 2012), breast cancer (Xiong et al., 2020), and papillary thyroid carcinoma (Gao et al., 2021). Protein, encoded by the *FKBP5* gene, is responsible for stress and metabolic-related disorders, including cancer (Barge et al., 2021). The association of *AR* gene and *FKBP5* gene as independent prognostic indicators for EAC has been shown (Smith et al., 2016). Given these circumstances, the above associations of piRNA and candidate EAC genes recommended for diagnosis of this disease should be considered as probable molecular markers.

Based on these results, several combinations of piRNA and candidate genes can be recommended for the diagnosis. The least expensive way is to use associations of one piRNA and one candidate gene, such as piR-65906 and the *FKBP5* gene, or the *LEP* gene and piR-73272, piR-77917, piR-78890. Another way is to use multiple piRNAs and two candidate genes, such as eight piRNAs and the FKBP5 and *SMAD4* genes. Another way to diagnose is to control many piRNAs (piR-5299, piR-5300, piR-5303, piR-5358, piR-6236, piR-7637, piR-19207, piR-34287, piR-65119, piR-89432, piR-92948, piR-94621, piR-95442, piR-96686, piR-100284, piR-101948, piR-108567, piR-127960, piR-151720, and piR-197022) in combination with several candidate genes in EAC (*ERBB3, FKBP5, LEP*, and *SEPP1*), which is certainly more appropriate, but more effective because it covers many piRNAs and candidate genes capable of causing EAC.

## Data Availability

The original contributions presented in the study are included in the article/Supplementary Material, further inquiries can be directed to the corresponding author.
