# Supplementary material for: piRNAs may regulate expression of candidate genes of esophageal adenocarcinoma
Source: Front Genet. 2022 Nov 30;13:1069637. doi: 10.3389/fgene.2022.1069637 (PMC9747755; doi:10.3389/fgene.2022.1069637)
Supplement: Supplementary file 1 [file Table1.PDF]

Supplementary Table S1. Esophageal adenocarcinoma candidate genes

Gene	PMID	Gene	PMID	Gene	PMID
ALDH1A2	25447851	*ERBB2*	24151090	*NOX5*	26901778
APOBEC1	25085003	*ERBB3**	24151090	*OXT*	26406593
*AR**	26467701	*ESR1*	26406593	*OXTR*	26406593
ARID1A	28440661	*FKBP5**	26467701	*PARP1*	23757351
AXIN2	26297437	*FOXF1*	26383589	*ROCK2*	26901778
BARX1	26383589	*FOXM1*	25889361	*RUNX3*	25229459
*BTG3**	25701359	*FOXP1*	26383589	*SEPP1**	22715394
*CD55**	26202380	*GDF7*	26783083	*SMAD4 **	24952744
CDK9	28404924	*GPBAR1*	28293080	*SOX2*	28692180
CDKN2A	25280564	*IGFBP2*	26317790	*TBX5*	26783083
CEP72	27527254	*LEP**	24569475	*TP53**	26733670
CTSE	25348778	*LGALS9*	28586026	*VDR*	25910066
DKK3	26093488	*MUC1*	28212575		
Note: * – indicates mRNAs, that are targets for piRNA with chosen criteria					

Supplementary Table S2. Characteristics of piRNA nucleotide interaction with CDS mRNA of the *AR* gene.

piRNA	Binding site, nt	ΔG, kJ/mol	ΔG/ΔGm, %	piRNA length, nt
piR-32860	1287	-127	92	24
piR-32860	1290	-127	92	24
piR-32860	1293	-127	92	24
piR-32860	1296	-127	92	24
piR-32860	1299	-127	92	24
piR-32860	1302	-127	92	24
piR-32860	1305	-127	92	24
piR-32860	1308	-127	92	24
piR-32860	1311	-127	92	24
piR-32860	1314	-127	92	24
piR-32860	1317	-127	92	24
piR-32860	1320	-127	92	24
piR-32860	1323	-127	92	24
piR-32860	1326	-127	92	24
piR-32860	1329	-127	92	24
piR-32860	2486	-123	89	24
piR-32860	2489	-123	89	24
piR-32860	2492	-123	89	24
piR-32860	2495	-123	89	24
piR-32860	2498	-123	89	24
piR-32860	2501	-123	89	24
piR-32860	2504	-123	89	24
piR-32860	2507	-123	89	24
piR-32860	2510	-123	89	24
Note. 5′UTR (1-1115 nt), CDS (1116-3878 nt), 3′UTR (3879-4315 nt)				

Note: The piRNA binding sites of different clusters in 5′UTR, CDS, and 3′UTR mRNA are colored in different shades of the same color, brown, blue, and green, respectively, in Supplementary tables S2 to S11. The interaction characteristics of piRNA with mRNA with a ΔG value of -170 kJ/mol or more are colored yellow. The values of ΔG/ΔGm equal to 99% and 100% are highlighted in red.

Supplementary Table S3. Characteristics of piRNA nucleotide interaction with CDS and 3′UTR mRNA of the *BTG3* gene.

piRNA	Binding site, nt	ΔG, kJ/mol	ΔG/ΔGm, %	piRNA length, nt
piR-75778	573	-140	99	27
piR-79273	578	-146	91	30
piR-61008	583	-134	94	27
piR-95539	586	-138	96	27
piR-68233	606	-168	94	33
piR-91587	610	-132	95	26
piR-82174	611	-151	95	29
piR-125506	618	-142	97	26
piR-89068	621	-178	92	34
piR-105455	621	-151	92	30
piR-36327	624	-140	92	27
piR-12399	626	-151	95	28
piR-37172	626	-151	90	31
piR-126527	626	-134	93	26
piR-129369	626	-149	92	29
piR-30978	627	-136	91	27
piR-124351	627	-138	94	26
piR-39214	629	-144	92	27
piR-110748	630	-155	92	29
piR-115618	630	-146	90	29
piR-33972	631	-140	94	27
piR-68075	632	-142	92	27
piR-98659	656	-155	91	31
piR-50295	657	-157	94	30
piR-89607	657	-125	92	26
piR-70728	661	-140	90	28
piR-40018	665	-138	93	26
piR-65262	1420	-144	92	29
piR-49502	1433	-140	90	29
piR-83600	1435	-172	92	34
piR-99370	1436	-134	90	28
piR-112090	1441	-153	91	31
piR-57784	1446	-146	90	31
piR-110734	1446	-163	90	34
piR-30958	1451	-146	90	30
piR-68737	1454	-144	92	29
piR-40345	1455	-157	95	31
piR-85280	1458	-130	94	26
Note. 5′UTR (1-260 nt), CDS (261-1151 nt), 3′UTR (1152-1595 nt)				

Supplementary Table S4. Characteristics of piRNA nucleotide interaction with CDS mRNA of the CD55 gene

piRNA	Binding site, nt	ΔG, kJ/mol	ΔG/ΔGm, %	piRNA length, nt
piR-112090	1420	-153	91	31
piR-110734	1433	-163	90	34
piR-65262	1435	-144	92	29
piR-49502	1436	-140	90	29
piR-83600	1441	-172	92	34
piR-99370	1446	-134	90	28
piR-57784	1446	-146	90	31
piR-30958	1451	-146	90	30
piR-68737	1454	-144	92	29
piR-40345	1455	-157	95	31
piR-85280	1458	-130	94	26
Note. 5′UTR (1-294 nt), CDS (295-1617 nt), 3′UTR (1618-2915 nt)				

Supplementary Table S5. Characteristics of piRNA nucleotide interaction with the 3'UTR mRNA of the *ERBB3* gene.

piRNA	Binding site, nt	ΔG, kJ/mol	ΔG/ΔGm, %	piRNA length, nt
piR-67286	4940	-151	92	31
piR-19207	4941	-146	91	29
piR-89432	4941	-168	90	33
piR-111933	4943	-140	90	29
piR-30807	4944	-144	91	29
piR-92948	4947	-151	93	30
piR-95442	4948	-142	94	29
piR-101948	4948	-140	92	29
piR-106532	4948	-136	93	28
piR-197022	4948	-136	90	29
piR-34287	4949	-149	91	31
piR-94621	4950	-151	91	30
piR-108567	4950	-161	95	32
piR-5300	4951	-149	95	30
piR-5303	4951	-146	92	30
piR-5358	4951	-149	91	30
piR-6236	4951	-151	93	30
piR-7637	4951	-151	93	30
piR-65119	4951	-142	92	29
piR-96686	4951	-146	92	30
piR-23387	4952	-161	95	31
piR-100284	4952	-157	94	31
piR-5299	4953	-138	93	28
piR-114722	4953	-130	90	28
piR-54974	4954	-138	90	28
piR-127960	4954	-136	91	28
piR-97970	5091	-144	91	29
piR-197190	5091	-144	91	30
piR-124546	5092	-155	90	31
piR-127715	5092	-168	90	34
piR-52164	5093	-153	91	31
piR-53915	5093	-136	94	27
piR-19207	5095	-151	93	29
piR-91087	5095	-144	93	30
piR-122261	5097	-127	92	26
Note. 5′UTR (1-276 nt), CDS (277-4305 nt), 3′UTR (4306-5758 nt)				

Supplementary Table S6. Characteristics of piRNA nucleotide interaction with the 3'UTR mRNA of the *FKBP5* gene.

piRNA	Binding site, nt	ΔG, kJ/mol	ΔG/ΔGm, %	piRNA length, nt
piR-65702	1382	-138	93	27
piR-125266	1389	-155	92	30
piR-119901	1394	-163	91	33
piR-91782	1395	-163	91	33
piR-74093	1402	-172	95	34
piR-38669	1402	-170	93	33
piR-55568	1403	-142	91	29
piR-122606	1404	-130	92	26
piR-49680	1404	-155	94	31
piR-21000	1405	-146	92	29
piR-15405	1408	-151	91	30
piR-15404	1409	-144	91	29
piR-68504	1415	-134	90	28
piR-51018	1415	-123	92	26
piR-97480	1418	-138	90	29
piR-143064	1419	-149	93	30
piR-99863	1420	-134	91	28
piR-14625	1420	-146	91	30
piR-65061	1422	-161	92	32
piR-56130	1422	-168	92	32
piR-123782	4854	-153	95	30
piR-195482	4860	-149	90	31
piR-117518	4861	-155	91	31
piR-102326	4862	-136	91	27
piR-92776	4862	-144	93	28
piR-62204	4862	-140	92	28
piR-75328	4862	-172	94	34
piR-56610	4862	-168	93	33
piR-115392	4863	-163	93	31
piR-93281	4863	-163	95	32
piR-38031	4864	-159	94	31
piR-101781	4868	-136	91	27
piR-62307	4868	-130	91	27
piR-74608	4868	-155	91	32
piR-56041	4868	-130	92	26
piR-35670	4870	-153	94	29
piR-125715	4871	-157	96	29
piR-72623	4872	-138	92	27
piR-136223	4875	-136	93	27
piR-91782	4940	-178	99	33
piR-198173	4943	-151	90	31
piR-91371	4946	-146	90	29
piR-74093	4947	-163	91	34
piR-49680	4949	-157	95	31

piR-21000	4950	-144	91	29
piR-15405	4953	-149	90	30
piR-114118	4962	-130	94	26
piR-97480	4963	-140	92	29
piR-99863	4965	-132	90	28
piR-150140	6356	-153	94	30
piR-72096	6356	-155	91	31
piR-195482	6357	-157	95	31
piR-70120	6357	-146	90	30
piR-117518	6358	-166	97	31
piR-102326	6359	-140	94	27
piR-92776	6359	-142	92	28
piR-62204	6359	-144	94	28
piR-75328	6359	-176	97	34
piR-56610	6359	-178	99	33
piR-115392	6360	-172	98	31
piR-93281	6360	-166	96	32
piR-43757	6361	-166	93	31
piR-38031	6361	-161	95	31
piR-85701	6362	-146	96	27
piR-101781	6365	-140	94	27
piR-74608	6365	-155	91	32
piR-35670	6367	-153	94	29
piR-125715	6368	-153	94	29
piR-72623	6369	-138	92	27
piR-77477	6371	-159	90	32
piR-91277	6373	-144	94	28
piR-92690	6373	-149	90	30
piR-74077	6374	-170	93	33
piR-56953	6377	-153	95	30
piR-1248	6378	-142	91	29
piR-10886	6379	-157	93	30
piR-83321	6381	-127	90	28
piR-138470	6386	-130	94	26
piR-59306	6390	-146	90	30
piR-65161	6393	-151	92	29
piR-102738	6397	-138	93	28
piR-84920	6397	-144	92	29
piR-50827	6397	-138	97	27
piR-57845	6397	-127	92	26
piR-36976	6397	-134	97	26
piR-104891	6398	-132	93	26
piR-107204	6398	-146	95	28
piR-19076	6399	-146	90	29
piR-59667	6404	-153	94	30
piR-9059	6404	-155	92	30

piR-73643	6408	-149	92	29
piR-82774	6410	-163	92	33
piR-64578	6414	-138	90	28
piR-88365	6421	-166	90	33
piR-94766	6423	-153	96	30
piR-110734	6429	-176	97	34
piR-57784	6429	-146	90	31
piR-72957	6430	-153	92	31
piR-30958	6434	-146	90	30
piR-30714	6435	-146	95	29
piR-32594	6435	-132	94	26
piR-23750	6436	-146	95	29
piR-30713	6436	-134	90	28
piR-68737	6437	-144	92	29
piR-59713	6437	-138	96	27
piR-53090	6438	-149	91	30
piR-197774	6439	-168	92	34
piR-112863	6439	-136	90	29
piR-161121	6440	-166	92	32
piR-123162	6441	-159	90	33
piR-85749	6441	-130	92	26
piR-5505	6441	-161	96	30
piR-156470	6442	-161	90	31
piR-112665	6442	-155	91	30
piR-67883	6443	-166	91	33
piR-30937	6445	-149	93	29
piR-104060	6446	-136	93	26
piR-111628	6446	-149	90	30
piR-2344	6446	-146	91	30
piR-79479	6449	-144	92	29
piR-15406	6450	-157	96	30
piR-109692	6452	-142	92	27
piR-20572	6459	-155	91	31
piR-67741	6460	-151	90	31
piR-51083	6463	-134	90	28
piR-68379	6464	-134	91	28
piR-33871	6464	-134	91	27
piR-96587	6522	-155	92	31
piR-48783	6522	-136	94	26
piR-128084	6524	-144	91	29
piR-110973	6527	-149	95	29
piR-99501	6529	-144	93	29
piR-48382	6529	-146	97	28
piR-59915	6529	-166	99	31
piR-12340	6529	-149	96	28
piR-35260	6530	-176	95	34

piR-159973	6532	-153	92	29
piR-116480	6532	-149	95	29
piR-103308	6532	-134	93	26
piR-55529	6532	-170	95	34
piR-81620	6533	-159	90	33
piR-131321	6538	-168	93	33
piR-197260	6539	-161	93	32
piR-87235	6539	-140	96	27
piR-94289	6540	-178	92	34
piR-62773	6540	-151	92	30
piR-78258	6540	-153	91	31
piR-196859	6542	-170	91	32
piR-54662	6543	-149	92	29
piR-85057	6544	-136	91	27
piR-37631	6547	-142	92	28
piR-55582	6548	-144	96	27
piR-68504	6575	-136	91	28
piR-51018	6575	-132	98	26
piR-114118	6577	-127	92	26
piR-97480	6578	-140	92	29
piR-106574	7100	-149	90	30
piR-197190	7101	-153	96	30
piR-93553	7101	-149	91	29
piR-96200	7101	-163	94	32
piR-97970	7101	-146	92	29
piR-99317	7101	-142	92	28
piR-127715	7102	-172	92	34
piR-97348	7102	-155	91	31
piR-41641	7102	-138	94	27
piR-53915	7103	-136	94	27
piR-89432	7105	-174	93	33
piR-91087	7105	-140	90	30
piR-19207	7105	-153	95	29
piR-199381	7107	-161	92	33
piR-87904	7108	-151	91	31
piR-138333	7109	-132	94	26
piR-92948	7111	-155	96	30
piR-197022	7112	-140	93	29
piR-101948	7112	-144	94	29
piR-95442	7112	-146	97	29
piR-34287	7113	-153	94	31
piR-108567	7114	-166	97	32
piR-94621	7114	-155	94	30
piR-5300	7115	-153	97	30
piR-5303	7115	-151	95	30
piR-5358	7115	-153	94	30

piR-6236	7115	-155	96	30
piR-7637	7115	-155	96	30
piR-35905	7115	-123	92	26
piR-65119	7115	-146	95	29
piR-96686	7115	-151	95	30
piR-101606	7115	-123	92	26
piR-100284	7116	-155	92	31
piR-114722	7117	-134	93	28
piR-5299	7117	-142	96	28
piR-147578	7118	-159	91	31
piR-127960	7118	-134	90	28
piR-54974	7118	-142	93	28
piR-126347	7119	-127	94	26
piR-125281	7120	-144	93	29
piR-102504	7129	-142	97	27
piR-98045	7129	-168	93	32
piR-46830	7129	-136	93	28
piR-151720	7131	-172	90	34
piR-198479	7136	-136	97	26
piR-124517	7137	-136	94	26
piR-37486	7138	-134	94	26
piR-109419	7142	-168	93	32
piR-96810	7142	-157	96	29
piR-82609	7145	-140	90	28
piR-43250	7145	-132	93	26
piR-62174	7147	-161	90	32
piR-2790	7150	-155	92	31
piR-123273	7151	-176	95	34
piR-59828	7154	-153	94	31
piR-115141	7155	-140	90	29
piR-83042	7155	-136	94	26
piR-37138	7159	-151	91	30
piR-126711	7160	-153	96	30
piR-106904	7160	-178	93	34
piR-92821	7162	-166	94	33
piR-95238	7162	-170	92	33
piR-37678	7162	-176	94	34
piR-123924	7163	-174	95	33
piR-86666	7163	-134	95	26
piR-65906	7163	-183	98	34
piR-121964	7164	-168	92	34
piR-120553	7165	-174	94	34
piR-124728	7165	-142	91	29
piR-125135	7165	-155	92	31
piR-85874	7165	-155	95	29
piR-96225	7165	-134	95	26

piR-50951	7165	-151	92	30
piR-106475	7166	-161	97	31
piR-79631	7166	-170	94	33
piR-169382	7167	-163	95	32
piR-123755	7167	-146	93	28
piR-93145	7167	-134	94	26
piR-96134	7167	-151	97	29
piR-45567	7167	-151	99	28
piR-124810	7168	-134	94	26
piR-98746	7168	-170	93	34
piR-40170	7168	-138	94	27
piR-37919	7168	-149	95	28
piR-85187	7169	-157	95	30
piR-47836	7169	-144	97	28
piR-54790	7169	-127	92	26
piR-124237	7171	-166	95	33
piR-111585	7171	-142	93	28
piR-99729	7171	-138	92	28
piR-102574	7172	-168	94	33
piR-66125	7172	-144	97	27
piR-32847	7172	-157	90	32
piR-107766	7173	-166	94	33
piR-37731	7173	-155	91	31
piR-74937	7174	-136	93	27
piR-35823	7174	-136	93	27
piR-126135	7175	-146	93	29
piR-109086	7201	-146	95	31
piR-121371	7274	-132	95	26
piR-84577	7304	-136	94	27
piR-91461	7305	-132	93	26
piR-196861	7306	-146	96	28
piR-83452	7306	-172	94	32
piR-123461	7307	-168	96	32
piR-51190	7307	-144	94	27
piR-85290	7308	-138	94	26
piR-65117	7308	-151	93	28
piR-93360	7309	-142	94	27
piR-60634	7309	-140	97	26
piR-70085	7310	-130	94	26
piR-99848	7312	-138	94	27
piR-30374	7313	-153	94	30
piR-106067	7315	-142	93	28
piR-89965	7326	-178	95	34
piR-99615	7329	-138	96	26

Note. 5′UTR (1-158 nt), CDS (159-965 nt), 3′UTR (966-7385 nt)

Supplementary Table S7. Characteristics of piRNA nucleotide interaction with the 3'UTR mRNA of the *LEP* gene.

piRNA	Binding site, nt	ΔG, kJ/mol	ΔG/ΔGm, %	piRNA length, nt
piR-38639	3084	-166	93	32
piR-93553	3087	-155	95	29
piR-96200	3087	-157	90	32
piR-97970	3087	-153	96	29
piR-99317	3087	-142	92	28
piR-197190	3087	-146	92	30
piR-41641	3088	-138	94	27
piR-55670	3088	-163	93	32
piR-97348	3088	-161	95	31
piR-124546	3088	-159	93	31
piR-127715	3088	-172	92	34
piR-34016	3089	-138	93	27
piR-52164	3089	-153	91	31
piR-53915	3089	-136	94	27
piR-19207	3091	-153	95	29
piR-76909	3091	-125	92	26
piR-89432	3091	-174	93	33
piR-91087	3091	-140	90	30
piR-122261	3093	-130	94	26
piR-199381	3093	-170	96	33
piR-30807	3094	-144	91	29
piR-87904	3094	-153	92	31
piR-92948	3097	-153	95	30
piR-95442	3098	-144	96	29
piR-101948	3098	-149	97	29
piR-197022	3098	-144	96	29
piR-34287	3099	-157	96	31
piR-94621	3100	-159	96	30
piR-108567	3100	-163	96	32
piR-5300	3101	-151	96	30
piR-5303	3101	-149	93	30
piR-5358	3101	-151	92	30
piR-6236	3101	-153	95	30
piR-7637	3101	-159	99	30
piR-35905	3101	-127	95	26
piR-65119	3101	-151	97	29
piR-96686	3101	-155	97	30
piR-101606	3101	-127	95	26
piR-23387	3102	-170	100	31
piR-100284	3102	-159	95	31
piR-5299	3103	-146	99	28
piR-114722	3103	-138	96	28

piR-54974	3104	-146	96	28
piR-127960	3104	-138	93	28
piR-147578	3104	-163	94	31
piR-126347	3105	-132	97	26
piR-125281	3106	-149	96	29
piR-102504	3115	-136	93	27
piR-47982	3116	-157	92	31
piR-39121	3119	-170	98	31
piR-43148	3127	-142	94	27
piR-66717	3170	-157	91	32
piR-41884	3172	-149	93	30
piR-97487	3172	-174	95	33
piR-124937	3173	-161	94	31
piR-1748	3175	-157	99	30
piR-66000	3175	-149	97	28
piR-105645	3175	-168	95	34
piR-72206	3176	-144	93	28
piR-48289	3177	-153	96	30
piR-83268	3177	-149	96	29
piR-114383	3177	-155	91	34
piR-146022	3179	-146	92	31
piR-61958	3182	-166	97	34
piR-37947	3183	-144	93	31
piR-103223	3183	-132	91	28
piR-198489	3183	-125	91	27
piR-36591	3184	-146	91	32
piR-197809	3185	-142	94	31
piR-84328	3186	-144	96	31
piR-70005	3187	-151	91	33
piR-50281	3188	-127	92	28
piR-59388	3188	-155	95	33
piR-114599	3189	-127	91	28
piR-129083	3189	-144	93	32
piR-47457	3190	-121	92	27
piR-68943	3190	-127	92	28
piR-42630	3192	-134	97	28
piR-65303	3194	-142	93	31
piR-67999	3196	-119	97	26
piR-73272	3219	-183	93	33
piR-79953	3220	-138	92	26
piR-77917	3221	-183	95	34
piR-72556	3223	-155	92	29
piR-27851	3226	-149	93	29
piR-186941	3227	-168	92	33
piR-57411	3230	-151	97	26
piR-78890	3231	-189	98	34

piR-98149	3232	-157	95	30
piR-198681	3232	-151	95	28
piR-67068	3233	-151	92	29
piR-35634	3234	-134	94	26
piR-66610	3234	-144	96	27
piR-108387	3236	-146	95	27
piR-84559	3237	-146	93	29
piR-127486	3238	-146	93	28
piR-43009	3239	-157	92	31
piR-85455	3239	-144	94	28
piR-114783	3239	-132	94	26
piR-37365	3240	-149	93	30
piR-93174	3242	-155	91	32
piR-42937	3246	-161	92	33
piR-75363	3246	-149	93	29
piR-82717	3246	-140	90	28
piR-85775	3246	-161	94	30
piR-124403	3246	-166	92	34
piR-78667	3247	-157	94	29
piR-114982	3247	-151	92	30
piR-126251	3255	-153	91	31
piR-121371	3256	-127	92	26
piR-94632	3257	-149	91	29
piR-124959	3258	-140	96	28
piR-25981	3261	-142	93	29
piR-65724	3261	-153	94	30
piR-115315	3264	-140	93	28
piR-118317	3268	-166	94	32
piR-4612	3271	-151	91	31
piR-6092	3271	-153	90	31
piR-6105	3271	-157	91	31
piR-132199	3293	-140	92	28
piR-35088	3296	-136	91	27
piR-13641	3305	-140	92	28
piR-33438	3305	-142	94	28
piR-69044	3305	-153	95	30
piR-86807	3307	-140	93	28
piR-87066	3307	-170	95	33
piR-86844	3308	-149	92	30
piR-93298	3308	-151	95	29
piR-126272	3308	-155	92	31
piR-42651	3309	-155	94	30
piR-59140	3312	-157	96	30
piR-198581	3312	-134	91	27
piR-32846	3315	-140	92	28
piR-85482	3322	-168	93	33

piR-128792	3322	-144	93	28
piR-99625	3346	-142	92	30
piR-83325	3348	-149	90	34
piR-124545	3354	-117	92	27
piR-197502	3358	-121	90	28
Note. 5′UTR (1-57 nt), CDS (58-561 nt), 3′UTR (562-3428 nt)				

Supplementary Table S8. Characteristics of piRNA nucleotide interaction with the 5'UTR mRNA of the *SEPP1* gene.

piRNA	Binding site, nt	ΔG, kJ/mol	ΔG/ΔGm, %	piRNA length, nt
piR-110480	108	-142	92	27
piR-61105	108	-166	91	33
piR-93553	109	-146	90	29
piR-89368	110	-168	90	34
piR-52164	111	-151	90	31
piR-89432	113	-170	91	33
piR-19207	113	-149	92	29
piR-199381	115	-159	90	33
piR-87904	116	-153	92	31
piR-30807	116	-144	91	29
piR-92948	119	-149	92	30
piR-197022	120	-140	93	29
piR-101948	120	-144	94	29
piR-95442	120	-140	93	29
piR-34287	121	-153	94	31
piR-108567	122	-159	94	32
piR-94621	122	-155	94	30
piR-101606	123	-123	92	26
piR-96686	123	-151	95	30
piR-65119	123	-146	95	29
piR-35905	123	-123	92	26
piR-5300	123	-146	93	30
piR-5303	123	-144	91	30
piR-5358	123	-153	94	30
piR-6236	123	-149	92	30
piR-7637	123	-155	96	30
piR-100284	124	-155	92	31
piR-23387	124	-166	98	31
piR-114722	125	-134	93	28
piR-5299	125	-142	96	28
piR-147578	126	-166	95	31
piR-127960	126	-134	90	28
piR-54974	126	-142	93	28
piR-126347	127	-127	94	26
piR-125281	128	-151	97	29
piR-102504	137	-142	97	27
piR-98045	137	-168	93	32
piR-46830	137	-136	93	28
piR-151720	139	-172	90	34
piR-198479	144	-136	97	26
piR-124517	145	-136	94	26
piR-37486	146	-134	94	26
Note. 5′UTR (1-188 nt), CDS (189-1334 nt), 3′UTR (1335-2175nt)				

Supplementary Table S9. Characteristics of piRNA nucleotide interaction with the 3'UTR mRNA of the *SMAD4* gene

piRNA	Binding site, nt	ΔG, kJ/mol	ΔG/ΔGm, %	piRNA length, nt
piR-199134	4316	-166	90	34
piR-84204	4322	-161	90	33
piR-65161	4331	-146	90	29
piR-49060	4333	-153	87	32
piR-36976	4335	-127	92	26
piR-50827	4335	-132	93	27
piR-84920	4335	-142	91	29
piR-116971	4335	-144	89	30
piR-125671	4335	-178	97	33
piR-82439	4336	-183	99	34
piR-100395	4336	-155	99	29
piR-107204	4336	-144	93	28
piR-19014	4337	-146	93	29
piR-19076	4337	-153	94	29
piR-82088	4337	-142	92	28
piR-125598	4337	-174	94	33
piR-127154	4338	-153	91	29
piR-9059	4342	-161	96	30
piR-46548	4342	-161	92	31
piR-59667	4342	-159	97	30
piR-9036	4343	-149	92	29
piR-68273	4344	-136	93	27
piR-57774	4345	-140	94	27
piR-98281	4379	-170	89	34
piR-69311	4387	-144	96	28
piR-121109	4398	-140	93	28
piR-56309	4401	-159	93	31
piR-81517	4401	-142	91	28
piR-98951	4401	-136	91	27
piR-74913	4402	-157	91	30
piR-71354	4408	-155	90	31
piR-198931	4408	-155	95	30
piR-40132	4411	-146	91	28
piR-74106	4411	-142	94	27
piR-124970	4428	-151	95	32
piR-44248	4440	-125	94	27
piR-97285	4473	-161	90	32
piR-104258	4473	-168	90	33
piR-48783	4475	-134	93	26
piR-96587	4475	-153	91	31
piR-110973	4480	-146	93	29
piR-12340	4482	-146	95	28
piR-48382	4482	-144	96	28

piR-59915	4482	-157	94	31
piR-99501	4482	-142	92	29
piR-55529	4485	-163	92	34
piR-70055	4485	-149	90	30
piR-70055	4486	-159	90	33
piR-84833	4492	-159	99	31
piR-78258	4493	-151	90	31
piR-94289	4493	-176	91	34
piR-54662	4496	-146	91	29
piR-85057	4497	-140	94	27
piR-83728	4502	-172	92	34
piR-12399	4509	-146	92	28
piR-115618	4513	-146	90	29
piR-33972	4514	-136	91	27
piR-68017	4514	-146	91	30
piR-5482	4516	-136	93	27
piR-38669	4516	-163	90	33
piR-74093	4516	-166	92	34
piR-55568	4517	-144	92	29
piR-37520	4518	-157	96	30
piR-49680	4518	-149	90	31
piR-122606	4518	-132	94	26
piR-21000	4519	-153	96	29
piR-89673	4519	-144	97	28
piR-58129	4520	-172	96	32
piR-15405	4522	-157	95	30
piR-15404	4523	-155	97	29
piR-51018	4529	-125	94	26
piR-68504	4529	-136	91	28
piR-114118	4531	-127	92	26
piR-97480	4532	-146	96	29
piR-64871	4535	-140	93	29
Note. 5′UTR (1-538 nt), CDS (539-2197 nt), 3′UTR (2198-8773 nt)				

Supplementary Table S10. Characteristics of piRNA nucleotide interaction with the 3'UTR mRNA of the *TP53* gene

piRNA	Binding site, nt	ΔG, kJ/mol	ΔG/ΔGm, %	piRNA length, nt
piR-102738	2340	-134	90	28
piR-107204	2341	-140	90	28
piR-111518	2342	-159	89	31
piR-151601	2456	-153	91	30
piR-134281	2456	-157	92	31
piR-112987	2463	-153	94	31
piR-82912	2474	-153	96	29
piR-165106	2476	-138	90	28
piR-123528	2478	-159	97	30
piR-96113	2483	-136	91	27
piR-44748	2485	-142	92	29
piR-44007	2494	-130	92	26
piR-121076	2496	-146	95	28
piR-46552	2496	-151	95	29
piR-51105	2499	-136	94	26
piR-83728	2505	-172	92	34
piR-153923	2508	-161	87	34
piR-129369	2512	-155	96	29
piR-44059	2512	-170	94	33
piR-12399	2512	-144	91	28
piR-80990	2513	-161	90	33
piR-30978	2513	-136	91	27
piR-91371	2518	-146	90	29
piR-49680	2521	-151	91	31
piR-89673	2522	-138	93	28
piR-21000	2522	-149	93	29
piR-81358	2533	-155	90	32
Note. 3′UTR (1-197 nt), CDS (198-1238 nt), 3′UTR (1239-2647nt)				

Supplementary Table S11. piRNA interacting with mRNA of *ERBB3*, *FKFP5*, *LEP* and *SEPP1* genes

piRNA	*ERBB3*	*FKBP5*	*LEP*	*SEPP1*
piR-93553	+	+	+	+
piR-52164	+	+	+	+
piR-89432	+	+	+	+
piR-19207	+	+	+	+
piR-199381	+	+	+	+
piR-87904	+	+	+	+
piR-30807	+	+	+	+
piR-92948	+	+	+	+
piR-197022	+	+	+	+
piR-101948	+	+	+	+
piR-95442	+	+	+	+
piR-34287	+	+	+	+
piR-108567	+	+	+	+
piR-94621	+	+	+	+
piR-96686	+	+	+	+
piR-65119	+	+	+	+
piR-35905	+	+	+	+
piR-5300	+	+	+	+
piR-5303	+	+	+	+
piR-5358	+	+	+	+
piR-6236	+	+	+	+
piR-7637	+	+	+	+
piR-100284	+	+	+	+
piR-114722	+	+	+	+
piR-5299	+	+	+	+
piR-127960	+	+	+	+
piR-54974	+	+	+	+
